# Benefit of using interaction effects for the analysis of high-dimensional time-response or dose-response data for two-group comparisons

**DOI:** 10.1038/s41598-023-47057-0

**Published:** 2023-11-27

**Authors:** Julia C. Duda, Carolin Drenda, Hue Kästel, Jörg Rahnenführer, Franziska Kappenberg

**Affiliations:** Department of Statistics, TU Dortmund University, Vogelpothsweg 87, 44227 Dortmund, Germany

**Keywords:** Genomics, Biotechnology, Functional genomics

## Abstract

High throughput RNA sequencing experiments are widely conducted and analyzed to identify differentially expressed genes (DEGs). The statistical models calculated for this task are often not clear to practitioners, and analyses may not be optimally tailored to the research hypothesis. Often, interaction effects (IEs) are the mathematical equivalent of the biological research question but are not considered for different reasons. We fill this gap by explaining and presenting the potential benefit of IEs in the search for DEGs using RNA-Seq data of mice that receive different diets for different time periods. Using an IE model leads to a smaller, but likely more biologically informative set of DEGs compared to a common approach that avoids the calculation of IEs.

## Introduction

With the rapid developments in next-generation sequencing (NGS) technology in the last decades, analyses of gene expression data have become regular in many laboratories^1^. A common goal is to identify differentially expressed genes (DEGs) that are responsible for the observable differences between, e.g., groups of individuals with different treatments or genotypes. Many software applications became available to optimally extract information from the large amounts of experimental data^2^. The mathematics behind these algorithms and models is often complicated, which can lead to suboptimal data analysis from practitioners and bioinformaticians. The interaction effect (IE) between two or more factors of interest is a methodological aspect that is often not considered in analyses where it could be beneficial. IEs are well-known in statistical modeling but are often not used in practice. Properly including and interpreting an IE in gene expression data analyses can be challenging, and the possibility of using an IE is often overlooked. An obvious reason for not using IEs in DEGs analyses might be the complexity of the statistical models and their correct computational implementation.

In the literature, there are many application examples similar to the one we will use throughout the manuscript, where an IE was likely beneficial to find interesting DEGs, but not considered. For example,^3^ dealt with time-restricted feeding of mice to test whether it could prevent obesity. They used DESeq2^4^ and the design included several factors such as genotype, feeding group, and time. In this setting, combining different variables to explore the interaction between e.g. time and genotype could have led to other, potentially more interesting DEGs. In another example^5^ used four separate study groups to analyze the differences in heart failure in mice. They either received a standardized chow or a high-fat diet for 12 weeks, and either additionally received angiotensin II after 8 weeks or not. Here as well, analyzing the excluded interaction between diet and hormones could lead to additional interesting insights.

Examples with an IE included in the DEG analysis were provided by^6,7^. Sloley et al.^6^ studied the exposure to high-frequency head impacts in mice. They use the DESeq2 package and their design contains an IE of the two factors treatment and injury. Similar methods are used in^7^, in which mice were treated with acarbose at three independent study sites. Their model contains the variables treatment, sex, and the interaction between them.

In this work, we explain the use, interpretation, and potential benefit of using IEs in gene expression analysis to identify DEGs. The article equips practitioners with a less profound statistical background with the knowledge to decide if the use of an IE helps answer their research question. We therefore aim at keeping the level of mathematical complexity low, to reach a wider range of potential users. Mathematical details can be found in^8,9^. We illustrate, explain, and compare DEG analyses with and without IE using a gene expression data set from^10^, where mice were fed either an unhealthy or a healthy diet for 3 to 48 weeks.

The article is structured as follows. We first explain the IE from different perspectives. Then we conceptually compare the use of an IE with the common approach that avoids modeling of interaction w.r.t. the resulting DEGs. The two methods are applied to the data set at hand and the differences in the results are discussed and explained in detail.

## Material and methods

### Data

The data set was first presented by^10^, where mice were fed with two different diets over the course of 48 weeks. One diet was the high-fat or ‘Western’ diet (WD) and the control was a standard diet (SD). The nine analysis time points within the 48 weeks were week 3, 6, 12, 18, 24, 30, 36, 42 and 48. In total 79 samples (mice) were used. The gene expression data from 35,727 genes were measured using RNA-seq. After removing the weeks with no data from mice in one of the two groups, 64 samples from the weeks 3, 6, 30, 36, 42, and 48 were left. To focus on the explanatory aim, analyses were mostly restricted to the data of weeks 3 and 6. The sample sizes in the remaining weeks are 7, 5, 5, 7, 3, 5 for SD and 5, 5, 5, 5, 4, 8 for WD. Further pre-processing is explained in “Implementation”.

### Interaction effects explained

**Figure 1:**
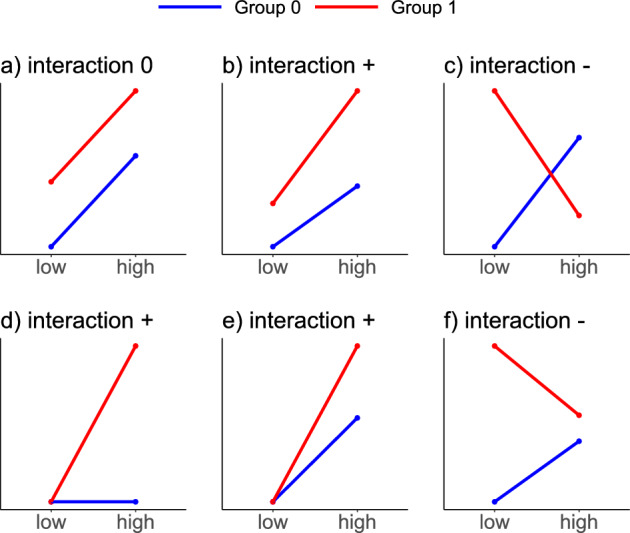
Schematic depiction of data scenarios without and with IE. (**a**) Group 0 (blue) and 1 (red) both have a positive effect for treatment high compared to low and a positive group effect, but no IE. (**b**) As in (**a**), but with an additional positive IE. (**c**) Negative IE between group and treatment. (**d**) No treatment effect for group 0. The treatment effect for group 1 is entirely represented by the IE. (**e**) Both groups display a positive treatment effect and there is no group effect in the treatment category low, only in high, i.e. an IE is present. (**f**) Negative IE between group and treatment, but no line crossing as in (**c**).

When two or more factors are of interest in an experiment, one should consider including IEs in the statistical model. Only using additive or main effects may not result in adequate modeling of the data. In Fig. 1, different effect scenarios are visualized using interaction plots for the case of two factors of interest, e.g. some group (0 = blue, 1 = red) and a compound with low and high concentration. In Fig. 1a, there is no interaction between the group and the concentration: The increase of the response from the low to the high concentration is the same for group 0 and group 1. At the same time, for a fixed concentration, the difference in the responses between group 0 and group 1 is the same. One can describe the *absence* of an IE graphically, biologically, and mathematically.

- Graphically, an additive effect or the lack of an IE results in parallel lines between the two groups.
- Biologically, the effect of the concentration does not interact with the effect of the group, because it is always the same increase in response from low to high concentration, regardless of the group.
- Mathematically, considering two factors with two levels each, a classical linear model, or equivalently an ANOVA model, with only additive effects for the two factors and normal noise is appropriate to model the data. This formalizes to
  1 $$\begin{aligned} y_j = \mu + \alpha \cdot g_j + \beta \cdot c_j + \varepsilon _j \end{aligned}$$
  where *j* indicates the sample, $g_j$ indicates if the *j*th sample is in group 0 ($g_j = 0$) or in group 1 ($g_j = 1$), and $c_j$ indicates if the *j*-th sample is exposed to the low concentration ($c_j = 0$) or the high concentration ($c_j = 1$).
  The mean difference in the responses for group 1 compared to group 0 is $\alpha$ and for increasing the concentration from low to high, the mean difference is $\beta$.
  For example, if the *j*-th sample is in group 0 ($g_j = 0$) and exposed to the low concentration ($c_j = 0$), the expected response is $\mu + 0 \cdot \alpha + 0 \cdot \beta$ = $\mu$. The intercept $\mu$ represents the mean response in the reference group (0) with the reference concentration (low).

The contrary case, the *presence* of a clear IE with a changed direction for the concentration effect, is depicted in Figure 1c. The crossing lines mean that the effect of a concentration increase is not additive (it is not the same within both groups). Instead, the concentration effect depends on the group, i.e. there is an *interaction* with the group effect. For group 0, an increase in the concentration leads to an increase in the response, whereas for group 1, an increase in the concentration leads to a decrease in the response. The additive model (1) can not capture this interaction as the model fit would force parallel lines into the effect plot. Mathematically, a model that accounts for the interaction between group and treatment is, therefore, an extension of the model in Eq. (1) by adding the IE $\gamma$:

2 $$\begin{aligned} y_j = \mu + \alpha \cdot g_j + \beta \cdot c_j + \gamma \cdot g_j\cdot c_j + \varepsilon _j. \end{aligned}$$

If the *j*-th sample is exposed to the higher concentration ($c_j=1$) and is in group 1 ($g_j = 1$), then the mean response is $\mu + \alpha + \beta + \gamma$. The interaction term $\gamma \cdot g_j\cdot c_j$ allows the lines in the interaction plot to be non-parallel. It is important to note that an IE does not necessarily visualize as a *crossing* of lines in an interaction plot, but simply *non-parallel* lines, such as in the examples shown in Fig. 1b, d, e, and 1f. We elucidate the use of IEs when analyzing real data in the context of biological research questions in “When do interaction effects capture the research question?”.

### Interaction effects calculated with DESeq2

In this section, we explain the mathematical background of gene expression modeling with the popular R-package DESeq2 ^4^. Details on statistical concepts presented here may not be relevant to readers who are more application-oriented and can be ignored without risking comprehension of the remaining sections. However, to understand an IE in more depth, we encourage to understand the parameters in the model formula (4).

Consider the count matrix *K*, where $K_{ij}$ are the count reads of gene *i* for sample *j*, $i \in \{1,...,n\}$, $j \in \{1,...,m\}$. To model the count data, DESeq2 uses a generalized linear model with a negative binomial distribution $K_{ij} \sim \text {NB}(\mu _{ij}, \tau _i)$ with mean $\mu _{ij}$ and gene-specific dispersion $\tau _i$.

The mean of the observed counts $\mu _{ij} = s_j q_{ij}$ is modeled with the parameter $q_{ij}$, which is proportional to the expected true concentration of fragments for sample *j* and rescaled with a sample-specific size factor $s_j$. The parameter $q_{ij}$ is modeled with a generalized linear model using the logarithmic link: $\text {log}_2(q_{ij}) = \sum _r \beta _{ir} x_{jr}$. In a factorial design, $x_{jr} \in \{0,1\}$ indicates if the *r*th explanatory variable applies to sample *j*, such that for the *i*th gene, $\beta _{ir}$ is the $\log _2$FC for factor level *r* compared to the reference factor level.

For our application example (“Data” ), the model has one factor for the diet (two values) and one factor for the week (six values). A model with the parameters for the week and diet without interaction is fitted for each gene *i*, $1 \le i \le 35,727$. In the following, we suppress the gene index *i* and consider the sample (mouse) index *j*. The model used in DEseq2 is then

3 $$\begin{aligned} \text {log}_2(q_{j}) = \mu + \alpha \cdot d_{j} + \sum _{r = 2}^6 \beta _{r}\cdot w_{jr}, \end{aligned}$$

where $\mu$ (intercept) denotes the response at the reference (SD and week 3), and $\alpha$ is the WD (main) effect. The variable $d_{j}$ is binary with value 0 for the SD and value 1 for the WD. The parameters $\beta _r$, $r\in \{2,...,6\}$, correspond to the week effects. The variable $w_{jr}$ is the indicator variable for the week, i.e. $w_{j2}=1$ only for week 6.

Now, adding an IE, the model is

4 $$\begin{aligned} \text {log}_2(q_{j}) = \mu + \alpha \cdot d_{j} + \sum _{r = 2}^6 \beta _{r}\cdot w_{jr} + \sum _{r = 2}^6 \gamma _{r}\cdot d_{j}\cdot w_{jr}. \end{aligned}$$

The parameter $\gamma _{2}$ denotes the IE between the factor diet and the factor week, comparing week 6 to week 3. The parameter $\gamma _{3}$ refers to the interaction between the diet and week, comparing week 30 to week 3, and so on. Due to the $\log _2$ transformation for the sample concentration $q_j$, the parameters must all be interpreted accordingly. For example, an IE of $\gamma _2=3$ means that the difference between the diet effect in week 3 and the diet effect in week 6 is $2^3=8$, or has a FC of 8.

### When do interaction effects capture the research question?

In RNA-Seq experiments, often the case of two factors, e.g. treatment and genotype, are analyzed, and it is of interest whether the effect of the treatment differs between the genotypes (in certain genes). The research question might be formulated as: Does the genotype affect the treatment effect? IEs capture such a research question and they should therefore be considered for the analysis.

In our application example, the two factors are diet and week, where diet is either a WD or a SD and week indicates the feeding duration. In this dataset measurements for different time points are available, and we focus on the two shortest durations, 3 weeks and 6 weeks, to explain the IE concept. The 3-week time point can be considered the reference level of the factor week. The research goal is to identify genes where activation/deactivation from weeks 3 to 6 induced by the WD is different compared to the SD. Mathematically, this research question translates into identifying genes with an IE between diet and week. Consequently, the use of a model that includes an IE should be considered.

### How do interaction effects capture the research question?

**Figure 2:**
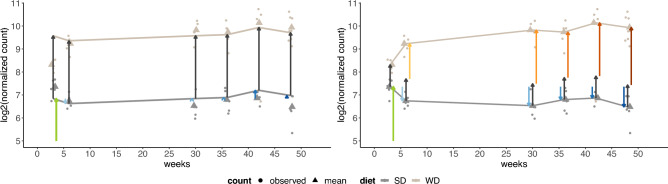
Visualization of the fitted model without IE (left) and with IE (right) for the mice dataset, for the gene identifier ENSMUSG00000069170 (Adgrv1). The arrows represent the estimated $\log _2$FCs according to Eq. (3) for the left fit, and Eq. (4) for the right fit. For both fits, $\mu$ (green arrow) is the expected mean gene expression level for the reference values three weeks and SD, and $\alpha$ (vertical dark grey arrows) is the estimated FC between SD and WD at each week. Further, both models include the week effects $\beta _r$ (blue arrows). The right model additionally includes interaction effects (yellow, orange, and red arrows) that correspond to $\gamma _r$ in formula (4).

To explain how IEs capture the research question, we visualize the benefit of adding IEs to a linear model, using our example dataset. In Fig. 2, for the mice groups, for each combination of diet type and week, expression values and fitted means are plotted, exemplary for one selected gene. Once no IEs are included in the model (Fig. 2, left), and once IEs are included (Fig. 2, right).

Without IEs, the estimated effect differences between the diets, represented by arrows, are mathematically forced to be the same across all weeks (vertical lines have the same length).

Consequently, in week 3, the effect is markedly overestimated, as the arrow between SD and WD is larger than the pure difference in group means. In contrast, if an IE is used (Fig. 2, right), then the group means estimated by the model capture well that the diet effect varies across weeks. The mathematical formulas of the estimated effects represented by the arrows are explained in “Interaction effects calculated with DESeq2”.

### Comparison of methods for estimating interaction effects

In this section, we compare the results obtained by fitting an interaction model between two factors (called Method II in the following) with a far more popular alternative, which we call Method I. The alternative approach avoids the direct modeling of an IE between two factors as follows: The data are split with respect to the second factor (e.g. week) into two groups $G_0$ and $G_1$. Then for group $G_0$ and $G_1$ separately, a model comparing the groups with respect to the first factor (e.g. diet) is fitted. Finally, it is analyzed, if for one group, typically the reference group $G_0$, no significant effect is observed, and for the other group $G_1$, there is a significant effect present.

The differences between the two approaches are illustrated and discussed on the mouse dataset, where for Method I the groups $G_0$ and $G_1$ are defined by week 3 (as reference) and week 6 (or larger week numbers, respectively). The models per week contain only one factor (diet) with two levels, SD and WD. Since separate models are fitted per week, the model-wise diet effect is allowed to vary across weeks.

When interpreting the results of the differential expression analysis, a consideration of both *statistical significance* and *biological relevance* is necessary: A *p*-value smaller than the significance level, which constitutes a statistically significant result, does not necessarily mean that the mean effect level, given here by the $\log _2$-Fold Change ($\log _2$FC), is of relevant size. On the other hand, a mean effect level larger than a pre-specified threshold, motivated by the biological context, does not always correspond to small *p*-values ^11^. Thus, to interpret a gene to be a differentially expressed gene (DEG), we always require two conditions to be fulfilled: The (FDR-adjusted) *p*-value is smaller than a significance level, and the $\log _2$FC is larger than a pre-specified threshold.

For the mouse dataset and the separate models (Method I), only those genes that show a diet effect (both significant and relevant) in week 6, but not in the reference week 3, are considered DEGs. The motivation is that interesting genes show no effect at the reference time point, where the diet had too little time to cause a differential effect, but later (at 6 weeks) the diet causes such a difference. For the interaction model (Method II), not two models but only a single model is fitted. To detect DEGs, one simply checks if the estimated IE is both significant and relevant.

- *Method I* (Separate): Separately for each week: Fit a one-factor model (two-group comparison, see equation (1)).
  A gene is DEG if the diet effect is both significant and relevant in week 6, but not both in week 3.
- *Method II* (Interaction): Fit a two-factor model between week and diet (including week, diet, and interaction), see equation (2).
  A gene is DEG if the IE is both significant and relevant.

**Figure 3:**
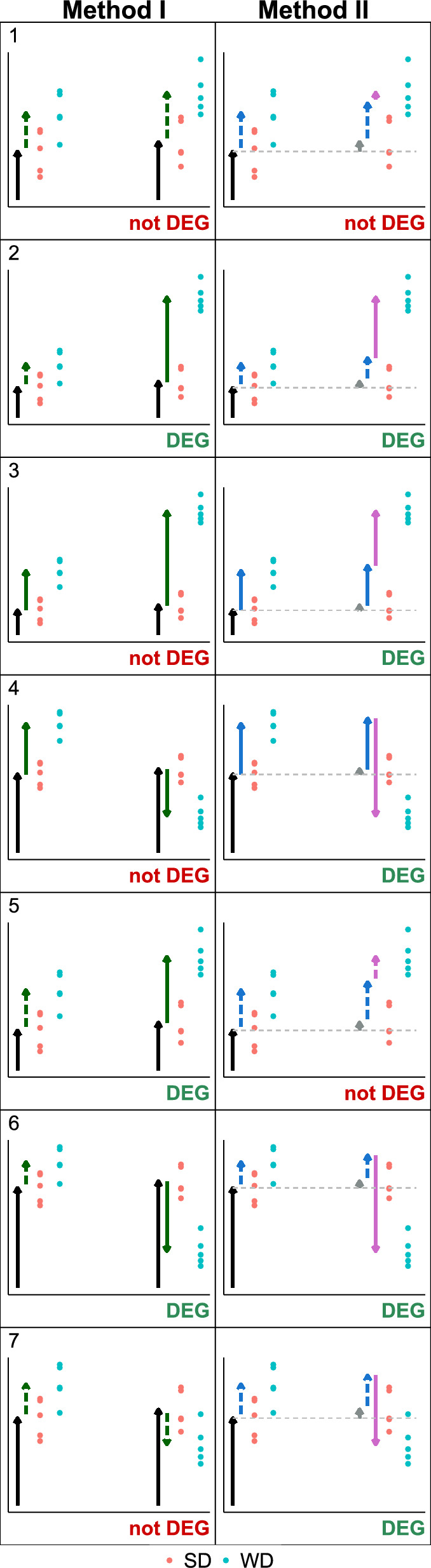
Visualization of seven example scenarios with different main effects and IEs, leading to different decisions for Method I (left column) and Method II (right column). Dots represent data points (blue: SD, red: WD; left: 3 weeks, right: 6 weeks), arrows represent effects (black: reference mean, green: main effect of diet, purple: IE). Dotted arrows indicate non-relevance (absolute effect size below threshold), solid arrows represent relevant effects. Dotted arrows are only shown for the main effects of IEs. The label ’DEG’ below a scenario indicates if the respective method classifies a gene as DEG (green) or not DEG (red).

To visualize the differences between the decision outcomes (gene is DEG or not) of Method I and II, Fig. 3 displays 7 cases using simulated data scenarios. The data are generated with constant residual variance, so that the decision is not influenced by differing variance values, but only by the estimated effect (arrow lengths).

- Case 1: Within both weeks, the estimated diet effect is not relevant (dotted green effect arrow). There is hence is no DEG by Method I. Since the effects are of similar size, the IE estimated by Method II (pink arrow) is not significant, and neither Method II classifies the gene as DEG.
- Case 2: In week 3, the effect is not relevant, in week 6 it is both significant and relevant. This leads to a significant IE for Method II. Therefore, both Method I and Method II classify the gene as DEG.
- Case 3: The diet effect is significant in both weeks. Since it is significant in week 3, Method I does not classify the gene as DEG. However, the diet effect in the second week is much larger, such that the IE is significant, and Method II classifies the gene as DEG.
- Case 4: Similar to case 3, but the effect direction of the diet effect changes: In the first week, there is a positive effect, and in the second week a negative effect. Again, only Method II classifies the gene as DEG.
- Case 5: In week 3, the diet effect is just below the significance level, whereas in week 6 it is just above the significance level. Therefore, Method I labels the gene as DEG. For Method II, the IE is not significant as the diet effect does not differ much between the weeks. Method II does not label the gene as DEG.
- Case 6: Similar to case 4, but the effect in week 3 is not significant. Now both methods classify the gene as DEG.
- Case 7: The direction of the diet effect changes. It is positive in week 3 and negative in week 6. Within each week, the effect size is not significant, therefore Method I classifies the gene as not DEG. The overall change in the effect represented by the IE is significant. Therefore, Method II labels this gene as DEG.

### Implementation

For all calculations, R^12^, version 4.2.2, and the packages DESeq2^4^, version 1.38.1, and topGO^13^, version 2.50.0, were used for determining DEGs and performing gene ontology enrichment analyses (GO EA), respectively. The entire code is shared on *GitHub* (https://github.com/jcduda/gene_expression_interaction). We specify the models of Method I and II in DESeq2 using

- Method I: DESeqDataSet(gse, design = $\sim$ diet)
- Method II: DESeqDataSet(gse, design = $\sim$ diet + weeks + diet:weeks)

In the example, the code for Method I is applied twice for separate weeks, i.e. for two different data sets ‘gse’, while the code for Method II is applied only once. Note that a model based on $\sim$ diet + weeks results in the same parameter values for each week, making it unsuitable for comparison with Method I and Method II, see Fig. 2.

One notable preprocessing step was the filtering. Removing only genes with less than ten counts over all samples resulted in a peak of the estimated diet effect at 0.206 (Supplementary Fig. 1). However, removing genes with more than 50% of samples with 0 counts leads to reasonably estimated effects without artifactual spikes in the histogram (Supplementary Fig. 2). Further, we shrunk the estimated effects using approximate posterior estimation with the *lfcShrink* function^14^. Effects that are non-zero only due to noise are shrunk to zero, while large, reliable effects are not affected.

**Figure 4:**
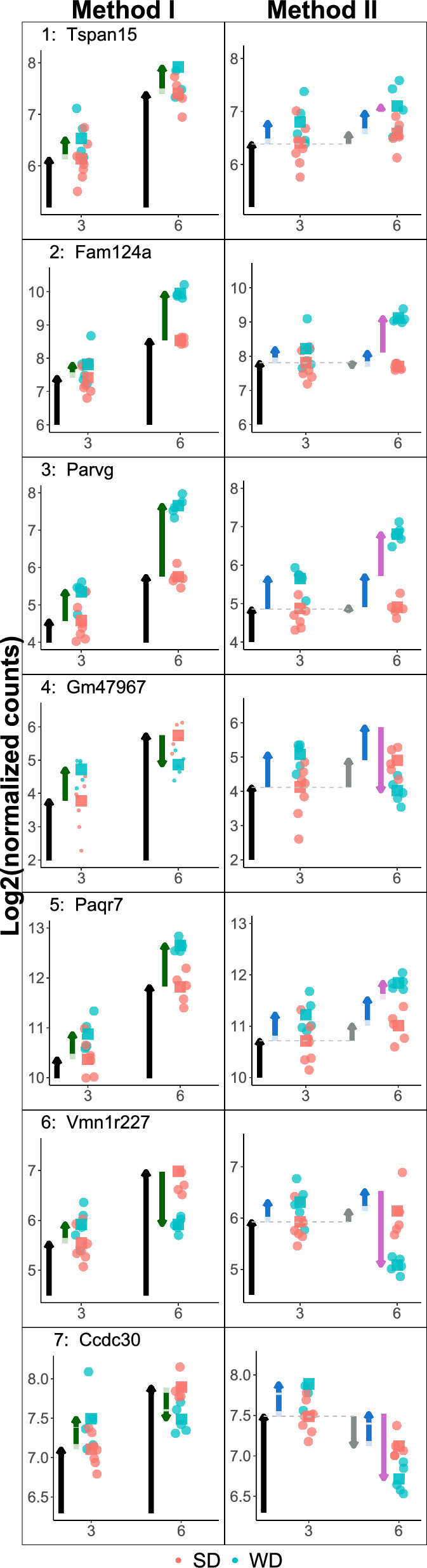
Example genes that are, according to DEG decision cases 1–7, not always classified in the same way by Method I (left) and II (right). Note that the original data are the same per gene (row), but due to the differences between Method I and II, background normalizations yield slightly different data for each gene. For normalization, DESeq estimates the library sizes as the median of the ratios of observed counts^9^. See caption of Figure 3 for an explanation of the arrows.

## Results

We compare Method I (separate) and Method II (interaction) for the mouse dataset, w.r.t. classification of genes as DEG or not DEG, as described in “Comparison of methods for estimating interaction effects”. In the following list, we define the terms significant, relevant, and DEG in the context of the example study.

For Method I we call a gene

- significant, if false discovery rate (FDR) adjusted *p*-value $< 0.05$ (for a specific week X)
- relevant, if absolute $\log _2$FC $> \log _2(1.5)$ (for a specific week X)
- DEG for week X, if it is significant and relevant for week X
- DEG, if it is not DEG for week 3, but DEG for week 6

For Method II we call a gene

- significant, if FDR adjusted *p*-value $< 0.05$ (for the IE)
- relevant, if absolute $\log _2$FC $> \log _2(1.5)$ (for the IE)
- DEG, if it is significant and relevant (for the IE)

For Method I, up-regulated DEGs for week X have a positive diet effect in week X. For Method II, up-regulated DEGs have a positive IE. Down-regulated DEGs are defined accordingly.

### Comparison of genes selected by Method I and Method II

We expect a relevant number of DEGs, since a biological effect of the diet (WD vs. SD) is reported by^10^. Table 1 shows the number of DEGs in week 3 and DEGs in week 6, according to Method I (simple comparison per week). There are more DEGs after 6 weeks of feeding compared to 3 weeks, both for up- or down-regulation. For up-regulated genes, 104 genes are DEGs only for week 3, 81 genes that are DEGs in both weeks, and 1,622 genes that are DEGs only in week 6. Hence, for Method I, regarding up-regulation, one would focus on the 1622 DEGs that are only identified for week 6 and not for week 3.

**Table 1:** Overview of DEGs for Method I, comparison of SD and WD.

	Week 3 only	Overlap	Week 6 only
Up	104	81	1,622
Down	81	93	726

Table 2 presents a main finding of our study, a comparison of DEGs identified with Method I and Method II. One can see that Method I (separate) identifies more DEGs than Method II (interaction). However, the DEGs identified by Method II are not all contained in the DEGs identified by Method I. There are almost 200 genes only identified by Method II, both for up-regulation and for down-regulation.

**Table 2:** Comparison of DEGs identified with Method I and Method II.

	Method I only	Overlap	Method II only
Up-regulated	914	695	167
Down-regulated	540	177	186

Note that 914 + 695 = 1609 does not equal 1622 in Table 1, because here we do not include genes that are downregulated in week 3, as otherwise they would not be DEG by Method I.

### Characterization of genes that are DEG only for Method I or only for Method II

To understand the benefits of the two methods, we characterize the genes that are only identified by one of the two approaches, respectively. After a mathematical characterization, we also investigate biological differences.

**Figure 5:**
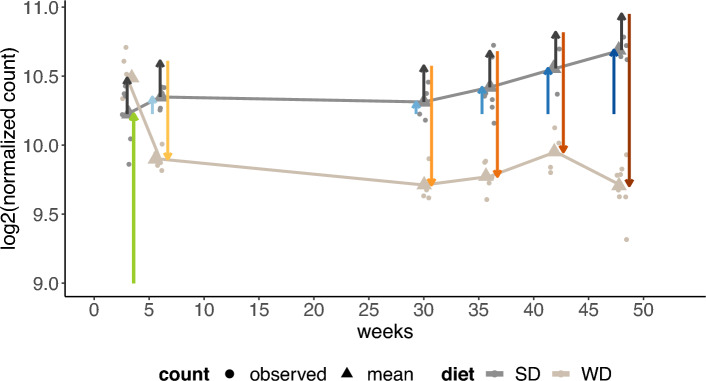
Expression pattern for the gene Sirt7, which is for the comparison week 3 vs. week 6 DEG for Method II (interaction), but not by Method I (separate), since the effect size is too low for week 6. See caption of Fig. 2 for detailed explanation of the arrows.

An insightful example is gene Sirt7 in Fig. 5, which is a typical case for being DEG by Method II, but not by Method I. From week 3 to week 6, there is an interaction between the factor week and diet (crossing of grey lines). The IE (large yellow arrow) is significant and relevant, making this gene DEG for Method II. However, for Method I the $\text {log}_2$FC of the diet effect in week 6 is not large enough to pass the threshold of $\text {log}_2(1.5)$. Hence, Sirt7 is not identified as DEG by Method I, even though an important underlying diet effect dependent on the time seems reasonable. Such genes are overlooked by the popular Method I.

**Figure 6:**
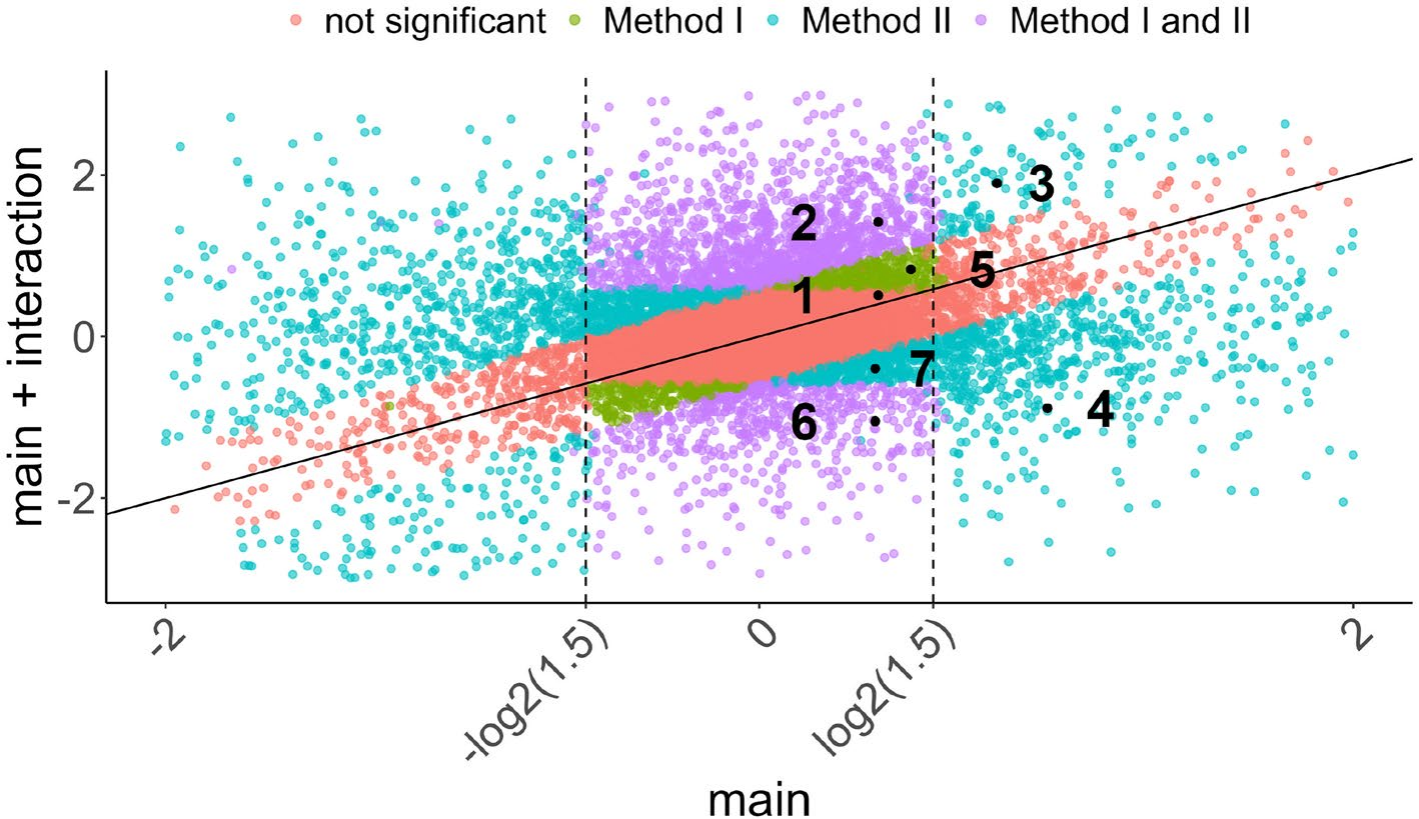
Characterization of regions of genes that are identified as DEG only by Method I or by Method II, or by both or none of the methods. The *x*-axis shows the estimated main effect (diet), i.e. the estimated $\log _2$FC from a SD to WD in the reference week 3, and on the *y*-axis the sum of this main effect and the IE, i.e. the overall effect between the two diets in week 6 in the interaction model, is plotted.

To better understand the differences between the two approaches, Fig. 6 shows regions of genes classified as DEG by both, none, or only one of the two methods, dependent on the main effect (diet) and the IE, as obtained by the interaction model (2) used by Method II.

Each dot represents a single gene. If there is no interaction (cf. Fig. 1a), the estimated IE is (close to) 0, such that the *x*- and *y*-value are identical and the gene is on the diagonal. For better illustration, the estimated effects are not shrunk and the decision rule depends on the $\log _2$FC threshold only. In practice, $\log _2$FC estimates should be subject to shrinkage and the classification into a DEG depends on both, $\log _2$FC and adjusted *p*-value (Supplementary Fig. 3 in the Appendix).

The genes can be divided into four groups according to the DEG classification of Method I and Method II. The numbers 1–7 assigned to regions match the simulated cases in Fig. 3 and a real gene expression pattern of a representative gene shown in Fig. 4. In the following, the gene expression patterns corresponding to the colored regions in Fig. 6 are explained.

- *Orange: not DEG for both methods.* Genes closer to the diagonal than $\log _2$(1.5), such that the IE is below this threshold and the gene is not DEG for Method II. Further, genes with absolute main effect above $\log _2$(1.5) are DEG for week 3 and thus not DEG for Method I.
- *Green: DEG only for Method I.* Genes with absolute main effect and IE less than $\log _2$(1.5), but overall effect in week 6 greater than $\log _2$(1.5). These genes are not DEG in week 3 by being slightly below the threshold but are DEG in week 6 by being slightly above the threshold. Hence, they are DEG for Method I, but the IE is small and the gene is not DEG for Method II.
- *Purple: DEG for Method I and II.* Genes with an estimated main effect (for week 3) below the $\log _2$FC boundaries, but the sum of main and IE (diet effect for week 6) is outside these boundaries. Hence, these genes are DEG for Method I. For Method II, they are DEG since the IE is large enough (points far from the diagonal line).
- *Blue: DEG only for Method II.* Genes that are not DEG for Method I since they are either DEG in week 3 (main effect outside $\pm \log _2$(1.5)) or have a main effect inside $\pm \log _2$(1.5) (as gene 7) but are not DEG in week 6, since the corresponding effect (main plus IE) is also within $\pm \log _2$(1.5)).

We further looked at differences concerning the biological conclusions of the found DEGs. First, a qualitative, small literature research on the top 10 (lowest adj. p-value) upregulated DEGs found only by Method I or only by Method II, respectively, suggests that both methods find genes that are reasonably associated with liver disease induced by a fatty diet (Table 4; Supplementary Table 1). On a broader scale, a GO EA was performed on the DEGs found by Method I, Method II, and the combination of both DEG sets (Table 3; Supplementary Table 2). Despite the smaller number of DEGs identified by Method II, the biological interpretation based on the processes identified by GO EA is very similar and plausibly covers immune activation related to fatty liver disease. This suggests that the DEGs found by Method II are more specific in the sense that they include fewer non-relevant genes while yielding similar GO EA results.

**Table 3:** Top 15 most significant GO groups found based on upregulated DEGs by Method I, Method II and combining the genes found by Method I and Method II. FDR-adjusted p-values are in parentheses.

	Method I	Method II	Method I or II
1	Immune system process ($2.44 \times 10^{-29}$)	Immune system process ($3.33 \times 10^{-28}$)	Immune system process ($2.27 \times 10^{-29}$)
2	Immune response ($2.44 \times 10^{-29}$)	Immune response ($3.33 \times 10^{-28}$)	Immune response ($2.27 \times 10^{-29}$)
3	Defense response ($2.44\times 10^{-29}$)	Cell activation ($3.33 \times 10^{-28}$)	Defense response ($2.27\times 10^{-29}$)
4	Pos. reg. of immune system process ($2.44\times 10^{-29}$)	Response to external stimulus ($5\times 10^{-28}$)	Regulation of immune system process ($2.27\times 10^{-29}$)
5	Regulation of immune system process ($2.44 \times 10^{-29}$)	Defense response ($6\times 10^{-28}$)	Pos. reg. of immune system process ($2.27\times 10^{-29}$)
6	Response to other organism ($2.44 \times 10^{-29}$)	Response to stimulus ($1.65 \times 10^{-27}$)	Response to external stimulus ($2.27 \times 10^{-29}$)
7	Response to external biotic stimulus ($2.44 \times 10^{-29}$)	Leukocyte activation ($2.57\times 10^{-27}$)	Response to biotic stimulus ($2.27\times 10^{-29}$)
8	Response to biotic stimulus ($2.44\times 10^{-29}$)	Regulation of immune system process ($1.2\times 10^{-25}$)	Response to other organism ($2.27\times 10^{-29}$)
9	Response to external stimulus ($2.44\times 10^{-29}$)	Response to external biotic stimulus ($2.27\times 10^{-25}$)	Response to external biotic stimulus ($2.27 \times 10^{-29}$)
10	Defense response to other organism ($2.44\times 10^{-29}$)	Response to other organism ($2.27\times 10^{-25}$)	Defense response to other organism ($2.27 \times 10^{-29}$)
11	Innate immune response ($2.44 \times 10^{-29}$)	Response to biotic stimulus ($2.27 \times 10^{-25}$)	Biol. proc. involved in interspecies interaction btw organisms ($2.27\times 10^{-29}$)
12	Cell activation ($2.44\times 10^{-29}$)	Pos. reg. of immune system process ($2.92 \times 10^{-25}$)	Cell activation ($2.27 \times 10^{-29}$)
13	Biol. proc. involved in interspecies interaction btw organisms ($2.44\times 10^{-29}$)	Pos. regulation of multicellular organismal process ($4.31\times 10^{-25}$)	Pos. regulation of multicellular organismal process ($2.27\times 10^{-29}$)
14	Inflammatory response ($2.44\times 10^{-29}$)	Biol. proc. involved in interspecies interaction btw organisms ($8.57\times 10^{-25}$)	Inflammatory response ($2.27\times 10^{-29}$)
15	Pos. reg. of response to external biotic stimulus ($2.44\times 10^{-29}$)	Pos. reg. of response to stimulus ($2.93\times 10^{-22}$)	Innate immune response ($2.27\times 10^{-29}$)

## Discussion

Using an IE model with 2 factors (Method II) instead of two separate models with one factor each (Method I) clearly changes the set of DEGs found in a gene expression analysis. The set of DEGs found with Method II is usually smaller. A theoretical reason for this is that statistical inference that aims at detecting IEs is less powerful in the sense that the sample size must be four times larger to have the same power for detecting an IE than to detect a main effect^15,16^, p. 100f.

Further, a gene that just passed the thresholds for being DEG for the reference group, but just not for the other group, is DEG for Method I but usually not for Method II, and it is not a good candidate for a biologically meaningful statement. The resulting DEGs for Method II are smaller in number, but lead to equally reasonable biological findings based on enrichment analyses. A limitation of Method II is that a single model with two main factors and an IE can be more difficult to interpret correctly than two models with one factor each and no IE. Quantifying if the smaller set of DEGs found by Method II contains less irrelevant genes is difficult for several reasons. First, a literature search to determine if a gene is not reported within the context of liver disease is fruitless. Due to false positive results and extensive research in this area, almost any gene can be found as associated. Second, the data set at hand does not have a clean reference, because mice were already fed with HFD for three weeks in the reference group, instead of being fed for zero weeks. However, within the limits of this study, the conceptual reasoning and analyses of GO enrichment analyses suggest that gene sets identified by Method II are smaller but likely contain fewer irrelevant genes.

## Conclusion

An IE might often be an adequate translation of a biological research question into a statistical concept. However, this relationship might remain unnoticed due to a lack of expertise or reluctance to deviate from routines. In this work, we offer an extensive explanation of IEs and why they might be scientifically relevant in the context of detecting differentially expressed genes (DEGs) in gene expression analysis.

We compare the IE-based approach (Method II) with a popular alternative approach (Method I) that avoids the calculation of IEs. While Method I detects more DEGs, many of them might not be scientifically relevant, whereas the smaller set of DEGs found with Method II can be interpreted as more specific by having fewer irrelevant genes. We encourage researchers to clarify for each project if an IE is the accurate mathematical representation of the formulated research question and to use this concept when appropriate. Further, if the research goal is to identify a smaller gene set containing less irrelevant genes (less false positives), we encourage to use Method II. However, if the research goal is rather exploratory and more false positives are acceptable, we suggest to use Method I.

### Supplementary Information

Supplementary Figures.

Supplementary Table 1.

Supplementary Table 2.

## Data Availability

The analyzed data sets are publicly available at the SRA database with reference number PRJNA953810.

## Appendix

See Table 4.

**Table 4:** Top 10 most significant up-regulated genes found only by Method I or Method II, respectively.

DEG only in	Gene	Log2FC	FDR-adj. p	Literature
Method I	Acnat2 (ENSMUSG00000060317)	2.02	$<0.01$	Considered a candidate for specific metabolic processes within the Type I acyl-CoA thioesterase/acyltransferase gene family^17^
	Tlr12 (ENSMUSG00000062545)	1.45	$<0.01$	Signaling in chronic liver diseases via complex immune responses mediating hepatocyte^18^
	Fgf21 (ENSMUSG00000030827)	2.39	$<0.01$	Associated with development and progression of NAFLD^19^
	Tgfbi (ENSMUSG00000035493)	0.79	$<0.01$	Overexpression in mice resulted in an increased incidence of spontaneous tumors and N,N-diethylnitrosamine (DEN)-induced liver tumor nodules^20^
	Ehhadh (ENSMUSG00000022853)	0.90	$<0.01$	Associated with development of fatty liver disease in dairy cows^21^
	Hpgds (ENSMUSG00000029919)	2.41	$<0.01$	Overexpression associated with adipogenesis and increased insulin sensitivity^22^
	Slc17a4 (ENSMUSG00000021336)	1.14	$<0.01$	An intestinal organic anion exporter expressed predominantly in the pancreas, liver, colon, and small intestine^23^
	Lgmn (ENSMUSG00000021190)	0.76	$<0.01$	Elevated expression of LGMN is reported in the tumor cells of liver^24^
	Slc7a8 (ENSMUSG00000022180)	1.26	$<0.01$	Slc7a8 deletion is protective against diet-induced obesity^25^
	Pgm3 (ENSMUSG00000056131)	1.17	$<0.01$	Up-regulated in livers of high fat diet fed mice^26^
Method II	Mmp12 (ENSMUSG00000049723)	4.16	$<0.01$	Mmp12 is a matrix metalloproteinase and associated with liver disease and inflammation^27^
	Cyp2c38 (ENSMUSG00000032808)	2.47	$<0.01$	Cyp2c family up-regulated in NAFLD mouse model^28^
	Itgam (ENSMUSG00000030786)	2.92	$<0.01$	Increase in Itgam (aka Cd11b) in liver X receptors knockout mice^29^
	Adgrg2 (ENSMUSG00000031298)	2.76	$<0.01$	Found upregulated in cholestasis liver tissue compared to mildly damaged liver tissue^30^
	Nap1l1 (ENSMUSG00000058799)	0.71	$<0.01$	Tumor promoter in hepatocellular carcinoma^31^
	Gstm3 (ENSMUSG00000004038)	2.40	$<0.01$	Associated with acute-on-chronic hepatitis B liver failure^32^
	Myo1c (ENSMUSG00000017774)	0.61	$<0.01$	Upregulation associated with human chronic liver disease^33^
	Rac2 (ENSMUSG00000025003)	1.89	$<0.01$	Associated with NAFLD^34^
	Cyp2c39 (ENSMUSG00000025003)	2.65	$<0.01$	Cyp2c family up-regulated in NAFLD mouse model^28^
	Gm3776 (ENSMUSG00000111709)	3.11	$<0.01$	Gm3776, or glutathione *S*-transferase (GST) alpha 13, belongs to GST genes that are associated with liver disease^35^

## Code availability

The code is available on *GitHub* (https://github.com/jcduda/gene_expression_interaction).
