# Supplementary material for: Benefit of using interaction effects for the analysis of high-dimensional time-response or dose-response data for two-group comparisons
Source: Sci Rep. 2023 Nov 27;13:20804. doi: 10.1038/s41598-023-47057-0 (PMC10682470; doi:10.1038/s41598-023-47057-0)
Supplement: Supplementary file 1 — Supplementary Figures. [file 41598_2023_47057_MOESM1_ESM.pdf]

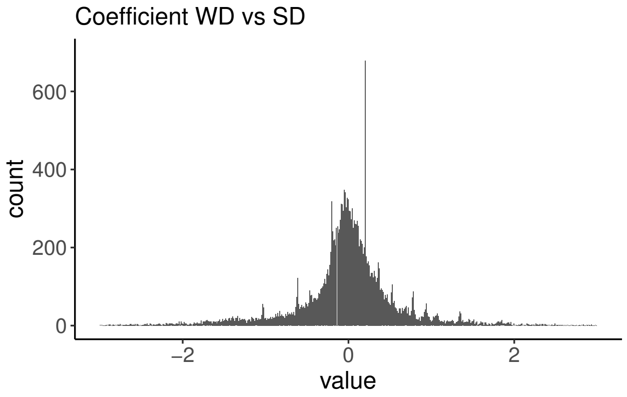

Figure 1: Histogram of the main effect (effect between SD and WD) in Method II with the filter "removing genes with less than ten counts over all samples".

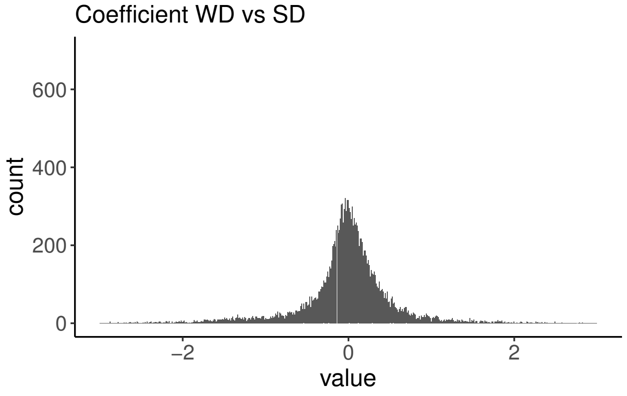

Figure 2: Histogram of the main effect (effect between SD and WD) in Method II with the filter "removing genes with more than 50% of samples with 0 counts".

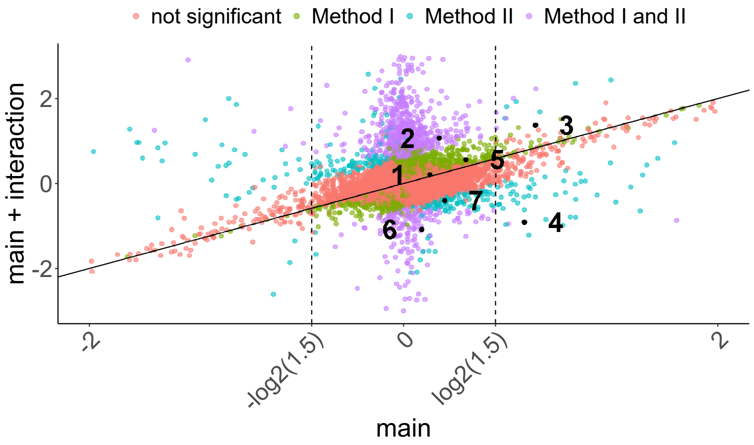

Figure 3: Characterization of regions of genes that are identified only by Method I or by Method II, or by both or none of the methods using p-values and $\log_2$FC. The x-axis is the estimated main effect of the factor diet in the interaction effect model of Method II with shrinkage. The y-axis is the sum of the main effect (x-axis value) and the interaction effect from the model in Method II with shrinkage. The main effect can be understood as the effect between SD and WD in week 3. The effect between SD and WD in week 6 is modeled as the respective effect in week 3 plus interaction effect.
